# Assessing Allele Frequency Information: A Study of Variant Curation Expert Panel Guidelines

**DOI:** 10.1155/humu/1611484

**Published:** 2026-04-10

**Authors:** Xiaoyan Wang, Tongmei Zhang, Yayun Qin, Yongyi Zou, Haiyan Luo, Haibo Li, Jieping Song

**Affiliations:** ^1^ Medical Genetics Center, Maternal and Child Health Hospital of Hubei Province, Wuhan, China; ^2^ Department of Histology and Embryology, School of Basic Medicine and Tongji Medical College, Huazhong University of Science and Technology, Wuhan, China, hust.edu.cn; ^3^ Department of Medical Genetics, Jiangxi Maternal and Child Health Hospital, Nanchang, China, jxsfybjyy.cn; ^4^ The Central Laboratory of Birth Defects Prevention and Control, Ningbo Women and Children′s Hospital, Ningbo, China

## Abstract

**Purpose:**

The 2015 guidelines recommend using a large, diverse, and race‐matched reference database. However, defining expectations in this context is subjective due to factors like genetic diversity and penetrance. ClinGen forms VCEPs to provide gene‐specific interpretations of ACMG/AMP guidelines, including population information. Our study evaluates VCEP guidelines on allele frequency information.

**Methods:**

We analyzed genetic codes in databases to determine the frequency and potential pathogenicity of variants among 39 VCEPs, considering factors like allele frequency thresholds and disease prevalence.

**Results:**

Our analysis found a variety of approved cutoffs among VCEPs, showing diverse disease mechanisms. We also noted variability in methods used to establish cutoffs and inconsistencies in parameters deemed necessary for approved thresholds.

**Conclusions:**

Understanding thresholds requires knowledge of genetics and diseases. VCEP guidelines on allele frequency evidence can help curators identify recommended thresholds. However, more guidance is needed for consistency in population evidence utilization.

## 1. Introduction

The standards and guidelines for interpreting genetic variants in clinical settings were collaboratively developed by ACMG/AMP (American College of Medical Genetics and Genomics/Association for Molecular Pathology) in 2015. The criteria provided defined how variants are classified as pathogenic (P), likely pathogenic (LP), variant of uncertain significance (VUS), likely benign (LB), or benign (B) according to specific evidence types, each with an assigned strength level [1]. Further guidelines were established to determine the specific combinations of criteria types and strengths necessary to classify variants as either P or B. Variants lacking adequate evidence were classified as VUS, creating a dilemma in clinical molecular genetic testing, as they should not be the sole basis for clinical decision‐making as per the guidelines established by the ACMG/AMP. Population data holds significant promise in assisting with variant classification, especially in the reclassification of VUS [2]. Population‐based guidelines BS1, BA1, and PM2 were created to set limits for understanding variants by comparing their allele frequency to the rarity of the related disorder [3]. Examining how often a genetic variant occurs in the overall population offers important information for determining its likelihood of causing disease, a process that can be done by referring to population databases that are available to the public. Per the criteria set forth by ACMG/AMP, if a variant is not found in a large control group (> 1000 individuals) of the same race as the patient with the variant, it could be seen as moderate evidence of pathogenicity (PM2). According to the guidelines established by ACMG/AMP, an allele frequency in a control population that exceeds the anticipated frequency for a particular disorder is indicative of strong evidence supporting a B interpretation for a rare Mendelian disorder (BS1). Allele frequency exceeding 5% is considered as standalone evidence (BA1) [4]. Nonetheless, numerous B variants are considered “private,” being unique to specific individuals or families, thereby rendering their absence in a population of the same race insufficient or weak evidence for pathogenicity. Population data is most useful for case–control comparisons when the populations are well characterized and show significant frequency differences and the Mendelian disease under investigation is known for early onset [5]. Nevertheless, the criteria for assessing these characteristics remain ambiguous, and the identification of suitable population evidence frequently necessitates a high level of expertise in the relevant gene and disease. ClinGen has created VCEPs in different disease areas of high importance, tasked with developing changes to the ACMG/AMP guidelines that are specific to the disease or gene they are focusing on [6]. These VCEP guidelines on population thresholds offer expert insights into the criteria necessary for utilizing population‐based evidence. The goal of this research was to clarify the key features of allele frequency thresholds that satisfy the BA1/BS1/PM2 standards through a comparison of VCEP suggestions. Thirty‐nine initial VCEPs have developed disease‐ and gene‐specific modifications to the ACMG/AMP guidelines, addressing a diverse array of conditions with different factors to consider [7–20]. Various metrics, such as inheritance pattern, disease prevalence, allelic contribution, and penetrance information, have been used to assess variants in the relevant genes, leading to a wide range of evaluations. The original population guidelines were used as a case study to assess the necessary parameters and level of evidence for each threshold. We thoroughly examined the main disease‐causing variants found by each VCEP in their guidance document while evaluating their initial variant classification to confirm the thresholds they established. This methodology facilitated an assessment of the degree to which the reported instances of variants adhere to the recommendations specified by the VCEPs and identified any discrepancies. The results of this research will be essential in pinpointing areas where further guidance is needed to ensure uniformity in variant classification, not only during VCEP recommendation creation but also in interpretation across clinical laboratories and other curation projects.

## 2. Methods

### 2.1. Assessment of ClinGen VCEP Guidelines

As of January 2026, 39 VCEPs that had endorsed and published variant interpretation guidelines were selected to evaluate their recommendations on the application of the BA1, BS1, and PM2 criteria. These 39 VCEPs cover all publicly available panels on the ClinGen official website (https://www.clinicalgenome.org/affiliates/variant-curation-expert-panels/), encompassing a broad spectrum of Mendelian disorders—including hereditary cancers (e.g., BRCA2, TP53, CDH1, and DICER1), neurodevelopmental conditions (e.g., SCN2A, RPGR, and Rett/Angelman‐like associated genes), metabolic diseases (e.g., GCK, ACADVL, and phenylalanine hydroxylase‐related disorders), rare myopathies (e.g., ACTA1, SGCB, and RYR1‐related malignant hyperthermia susceptibility [MHS]), X‐linked conditions (e.g., SLC6A8‐related creatine transporter deficiency and MECP2‐related Rett syndrome), and lysosomal storage diseases (e.g., GAA‐related Pompe disease). All cited literature contains detailed information on the clinical characteristics of the relevant diseases and specific adjustments to the ACMG/AMP recommendations.

When collating the initial recommendations, emphasis was placed on key criteria commonly mentioned by multiple expert panels, including heterozygosity, control populations, thresholds, and average coverage (Table 1). Furthermore, in light of the subsequent finding in the discussion that VCEPs differ in parameter settings, supplementary documentation was added regarding the sources of core parameters (e.g., disease prevalence, penetrance, allelic contribution, and genetic contribution) adopted by each VCEP. These parameters were categorized into “validated parameters” (derived from population cohorts, clinical registries, or functional experimental data) and “extrapolated parameters” (utilizing surrogate indicators or conservative assumptions due to limited data) (Table 1). Among the 39 VCEPs, 25 (62.5%) used fully documented validated parameters (e.g., the ABCA4 VCEP integrated a validated prevalence of 1 in 7500 for autosomal recessive retinopathy, and the GCK VCEP calibrated inputs using glucose tolerance test outcomes), while 15 (37.5%) resorted to extrapolation (e.g., the SLC6A8 VCEP used plasma creatine/creatinine ratios as a proxy for disease prevalence, and the RYR1 VCEP adopted a universal penetrance estimate of 0.5 for autosomal recessive variants) [12, 14, 18, 21]. The proportion of these two types of parameters was calculated, laying the foundation for the subsequent analysis of threshold heterogeneity.

**Table 1:** Source types and proportions of parameters in 40 VCEPs.

Parameter source type	Number of VCEPs	Proportion	Typical cases
Validated parameters (derived from population cohorts, clinical registries, or functional experimental data)	25	64%	ABCA4 VCEP (autosomal recessive retinopathy, with a validated prevalence of 1/7500); GCK VCEP (calibrated input data using glucose tolerance test results); MYOC VCEP (primary open‐angle glaucoma, integrated population‐based penetrance estimates and longitudinal disease progression data)
Extrapolated parameters (using surrogate indicators or conservative assumptions due to limited data)	14	36%	SLC6A8 VCEP (adopted plasma creatine/creatinine ratio as a surrogate for disease prevalence); RYR1 VCEP (used a universal penetrance estimate of 0.5 for autosomal recessive variants); AIPL1 VCEP (Leber congenital amaurosis, inferred allelic contribution using photoreceptor cell‐type expression data)

Notably, the Whiffin–Ware calculator emerged as a foundational tool utilized by 37 out of the 39 VCEPs (92.5%) to establish allele frequency thresholds for BA1, BS1, and PM2 [19]. Specific details regarding its application were integrated into this subsection: The calculator requires input of four disease‐specific parameters (disease prevalence, penetrance, maximum allelic contribution, and maximum genetic contribution), which are adjusted to balance the risk of misclassifying P variants as B and vice versa—ensuring the clinical relevance of the resulting thresholds. For the remaining three VCEPs (e.g., the RASopathy VCEP and the Brain Malformation [BM] VCEP focusing on AKT3/MTOR/PIK3CA/PIK3R2 somatic variants), alternative computational frameworks (e.g., Bayesian models incorporating gene‐specific contribution data and adjusted PM2 thresholds for cancer dataset contamination) were used [19], and these frameworks were also documented to ensure comprehensive coverage of threshold‐setting methodologies.

### 2.2. Variant Identification

To clarify how each of the 39 VCEPs utilizes population evidence during the variant curation process, the initial and final classifications (P, LP, VUS, LB, and B) of variants by each VCEP were first documented. Subsequently, for the initial variant interpretations that incorporated evidence from the BA1, BS1, and PM2 criteria, the exact thresholds referenced were accurately identified by reviewing VCEP publications, ClinVar (https://www.ncbi.nlm.nih.gov/clinvar/), and the ClinGen Evidence Repository (https://erepo.clinicalgenome.org/evrepo/) [8, 13, 19].

Considering the impact of database selection on thresholds (a key point in the Discussion section), supplementary records were added regarding the types of population databases (e.g., general population databases, e.g., the full gnomAD v4.0, and disease‐excluded databases, e.g., the gnomAD noncancer subset v3.1.2) and their version information used by each VCEP for threshold calibration [22, 23]. Among the 39 VCEPs, 33 (82.5%) relied on general population databases, while seven (17.5%)—primarily those focused on cancer predisposition genes (e.g., BRCA2, CDH1, and DICER1) and late‐onset disorders—prioritized disease‐excluded databases to reduce bias from undiagnosed cases [8, 9, 11]. Additionally, for VCEPs that excluded specific variant types (e.g., BRCA2 insertions/deletions, MSH2 initiation codon variants, and RYR1 splice site variants), detailed documentation was provided on the excluded variant types and the reasons behind the exclusions (e.g., poor sequencing quality, unique functional constraints, and high false‐positive calling rates), which were linked to the subsequent technical consideration analysis of threshold setting.

Beyond calculating the allele frequencies used as evidence in the initial variant interpretations, the thresholds mentioned by VCEPs when adjusting the criteria to support the acceptance or rejection of BA1, BS1, and PM2 were also recorded. Special attention was paid to whether the thresholds were modified to account for factors such as sequencing errors (e.g., minimum read depth ≥ 25 required by the CDH1 VCEP), clonal hematopoiesis (addressed by the DICER1 VCEP), and database contamination (mitigated by the PTEN VCEP via exclusion of bottlenecked populations) [8, 9, 16]. Concurrently, in line with the SVI (Sequence Variant Interpretation) Working Group′s recommendations (outlined in the Results), documentation was included on VCEPs′ adherence to allele count requirements: 38 out of 39 VCEPs (95%) mandated that variants must be present in at least five alleles among a minimum of 2000 tested alleles for BA1/BS1 eligibility (where feasible), while the remaining two VCEPs (focused on ultrarare disorders with small cohort sizes, e.g., the AIPL1 VCEP for Leber congenital amaurosis) adjusted this requirement to ≥ 3 alleles in ≥ 1000 tested alleles [24].

### 2.3. Assessment of BA1/BS1/PM2 Impact on Variant Classification

#### 2.3.1. Data Selection

As of January 2026, 9441 variants labeled as “accepted” were selected from the ClinGen Variant Curation Interface (a public genetic variant interpretation platform based on the ACMG/AMP guidelines) for the study. These variants were contributed by all 39 ClinGen VCEPs and categorized as follows: 1051 B, 960 LB, 2845 VUS, 1948 LP, and 2637 P. The expanded sample size reflects the increased volume of curated variants in the ClinGen database as of 2026 and ensures sufficient statistical power to analyze threshold variability across the 39 diverse VCEPs—including those focusing on rare subtypes (e.g., the ENG VCEP for hereditary hemorrhagic telangiectasia and the SERPINC1 VCEP for antithrombin deficiency) that contribute smaller but clinically critical variant sets.

#### 2.3.2. Assessment Methods

The classification system proposed by Richards et al. was adopted, and a Bayesian point–based system developed by Tavtigian et al. (and refined by the ClinGen SVI Working Group in 2025) was incorporated to quantify the impact of BA1, BS1, and PM2 on the final variant classifications [1, 20]. This updated point‐based system assigns scores ranging from +1 to +8 to P evidence and scores ranging from −1 to −8 to B evidence, with revised predefined cutoff values for each classification (B: ≤ −7; LB: −1 to −6; VUS: 0 to +5; LP: +6 to +9; and P: ≥ +10) to address previous limitations in distinguishing between LB/VUS and LP/VUS. The total score is mapped to the final classification using these updated cutoffs.

#### 2.3.3. Supplementary Analysis

Borderline variants were defined as those classified as VUS via the ACMG/AMP framework but with scores that were within 1 point of the LP cutoff or LB cutoff when BA1/BS1/PM2 were included. Among the 2094 VUS variants applied to the population data, 591 (28%) were identified as borderline—350 near the LP cutoff and 241 near the LB cutoff.

**Figure 1:**
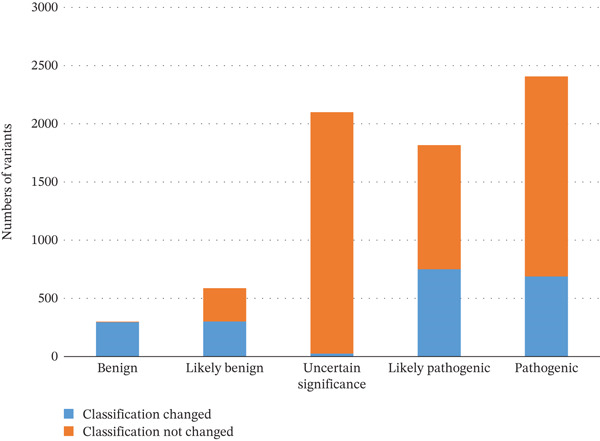
Change in the classification of variants after removal of BA1/BS1 scores.

Additionally, to evaluate the impact of reclassifying PM2 as a “corroborating strength” factor (as recommended by the 2025 SVI Working Group update), a simulation analysis was conducted. This involved removing the PM2 score and recording the proportion of LP variants that were downgraded to VUS: 41.2% (747 out of 1813) of LP variants were downgraded. Similarly, the proportion of LB variants that were downgraded to VUS after removing the BA1/BS1 scores was documented: 79.5% (302 out of 380) of LB variants were downgraded, with notable variation across disease areas (Figure 1).

**Figure 2:**
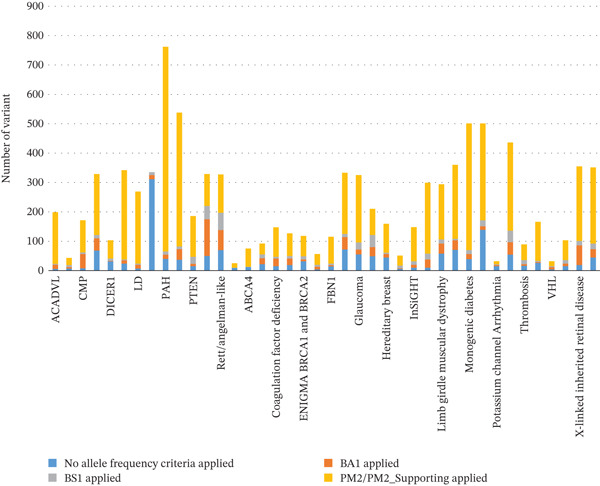
Number of variants incorporating BA1/BS1/PM2 for each classification.

Furthermore, given the observed variability in criterion utilization across the 39 VCEPs, supplementary statistical analyses were included. This involved calculating the percentage of variants incorporating BA1/BS1/PM2 for each VCEP: The highest utilization rates were observed in the Phenylketonuria VCEP (94.8%, 722 out of 762 variants), while the lowest rates were in the MHS VCEP (7.2%, 24 out of 335 variants) (Figure 2). These percentages were linked to the unique genetic and clinical characteristics of the diseases targeted by the respective VCEPs (e.g., the clear genetic basis of Pompe disease for the Lysosomal Diseases [LD] VCEP, the complex RYR1 variant classification—including incomplete penetrance and variable expressivity—for the MHS VCEP, and gene‐specific contribution variability for the RASopathy VCEP).

## 3. Results

### 3.1. Whiffin–Ware Calculator and SVI Working Group Recommendations

The Whiffin–Ware calculator is a foundational tool utilized by the majority of VCEPs to establish allele frequency thresholds for the three core population–based criteria: BA1, BS1, and PM2. Its application requires the input of four key parameters, each tailored to the unique genetic and clinical characteristics of the target disorder: disease prevalence (estimated occurrence in the general or affected population), penetrance (proportion of individuals with a variant who develop the disease phenotype), maximum allelic contribution (the highest proportion of disease cases attributed to a single variant), and maximum genetic contribution (the proportion of disease cases linked to the target gene overall) [19]. These parameters are adjusted to ensure thresholds are clinically relevant, balancing the need to avoid false classification of P variants as B and vice versa.

The SVI Working Group provided critical, cross‐cutting recommendations that guided threshold setting across multiple VCEPs, addressing limitations in the original ACMG/AMP framework:

1. PM2 reclassification: PM2 should be designated as a “corroborating strength” factor rather than a definitive criterion, acknowledging that variant rarity alone is not sufficient for pathogenicity but supports other evidence.
2. BS1 population stratification: BS1 should only be applied to populations with well‐characterized probands, as genetic variant prevalence often varies by ethnicity—particularly relevant given that many existing genetic datasets are biased toward Caucasian populations.
3. Allele count requirements: To minimize the impact of sequencing errors, calling artifacts, or accidental inclusion of affected individuals in control databases, variants must be present in at least five alleles among a minimum of 2000 tested alleles in a population group (where feasible) to qualify for BA1/BS1.
4. PM2 flexibility for low‐frequency variants: PM2 may be applied to variants detected at extremely low frequencies (not just complete absence) in large population databases (e.g., gnomAD), accounting for potential contamination from cancer datasets, clonal hematopoiesis–related variant enrichment, and inherent database inaccuracies.

### 3.2. Allele Frequency Thresholds for BA1/BS1/PM2 by VCEP

Table 2 presents the standardized parameters and final allele frequency thresholds for BA1, BS1, and PM2 across VCEPs. This tabular format enables direct comparison of threshold‐setting strategies, highlighting variations in parameter selection (e.g., prevalence and penetrance) and resulting AFTs.

**Table 2:** Allele frequency thresholds for BA1, BS1, and PM2 in VCEPs of different disease types.

VCEPs	Representative gene	BA1 threshold	BS1 threshold	PM2 threshold
CDH1	CDH1	0.002	0.001	0.00001
Epilepsy Sodium Channel	SCN2A	0.01	0.00002	0.0001
ACADVL	ACADVL	0.007	0.0035	0.001
Malignant Hyperthermia Susceptibility	RYR1	0.0038	0.0008	Not applicable
Lysosomal Diseases	GAA	0.01	0.005	0.001
Brain Malformations	AKT3	0.000926	0.000185	Variants present in ≤ 1 individual in gnomAD
Cardiomyopathy	MYH7	0.001	0.0002	0.00004
DICER1 and miRNA‐Processing Gene	DICER1	0.003	0.0003	0.000005
Familial Hypercholesterolemia	LDLR	0.005	0.002	0.0002
InSiGHT Hereditary Colorectal Cancer/Polyposis	APC	0.001	0.00001	Allele count is > 1 (0.000003); allele count is ≤ 1 (0.00001)
Phenylketonuria	PAH	0.015	0.002	0.0002
Platelet Disorders	ITGA2B	0.0024	0.00158	PM2 (absent); PM2_Supporting (0.0001)
PTEN	PTEN	0.00056	BS1 (0.000043 to 0.00056); BS1_Supporting (0.0000043 to 0.000043)	0.00001 or 0.00002 (multiple alleles)
RASopathy	SHOC2	0.0005	0.00025	Completely absent
Rett and Angelman‐like Disorders	UBE3A	0.0003	0.00008	Absent or zero observations
ABCA4	ABCA4	0.163	0.0163 or 0.00163	0.0001
Antibody Deficiencies	CTLA4	0.0000111	0.00000111	0.000000143
Coagulation Factor Deficiency	F8	0.000333	0.0000333	Absent in males
Congenital Myopathies	ACTA1	0.0025	0.00025	0.000005
ENIGMA BRCA1 and BRCA2	BRCA1	0.001	0.0001	Absent from controls
Epilepsy Sodium Channel	SCN8A	0.0001	0.000002	One or fewer alleles
FBN1	FBN1	0. 001	0.00005	0.000005
Glaucoma	MYOC	0.01	0.001	0.0001
Hearing Loss	CDH23	0.005	0.003 or 0.0007	0.00002
Hereditary Breast, Ovarian and Pancreatic Cancer	ATM	0.005	0.0005	0.00001
Hereditary Hemorrhagic Telangiectasia	ACVRL1	0.01	0.0008	0.00004
Leber Congenital Amaurosis/early onset Retinal Dystrophy	AIPL1	0.0057	0.00569	0.0004
Limb Girdle Muscular Dystrophy	ANO5	0.003	0.001	0.0001
Mitochondrial Disease Nuclear and Mitochondrial	mtDNA	0.01	0.005 ‐ 0.0099	0.00002
Monogenic Diabetes	GCK	0.0001	0.00004	0.000003
Myeloid Malignancy	RUNX1	0.0015	0.00015	0.00005
Potassium Channel Arrhythmia	KCNQ1	0.004	0.0004	0.00001
Severe Combined Immunodeficiency Disease	ADA	0.00721	0.00161	0.0001742
Thrombosis	SERPINC1	0.002	0.0002	0.0000
TP53	TP53	0.001	0.0003	0.00003
VHL	VHL	0.000156	0.0000156	0.00000156
von Willebrand Disease	VWF	0.1	0.01	0.0001
X‐linked Inherited Retinal Disease	RPGR	0.05	0.000083	0.00005

## 4. Key Observations Across VCEPs

### 4.1. Variability in Criterion Utilization

The application of population‐based criteria (BA1/BS1/PM2) varied substantially among VCEPs (Figure 2). LD and ACADVL VCEPs exhibited the highest utilization rates, with 262 out of 269 (97.4%) and 193 out of 199 (96.9%) variants, respectively, incorporating these criteria. This reflects the clear genetic basis of their target disorders (e.g., all Pompe disease cases linked to GAA variants), making allele frequency a robust classification tool. In contrast, the MHS VCEP rarely used BA1/BS1/PM2 (24 out of 335 variants, 7.2%) (Figure 2), due to the complexity of RYR1 variant classification—including incomplete penetrance, variable expressivity, and the presence of numerous known P variants in gnomAD (limiting PM2 utility) [13, 21].

**Figure 3:**
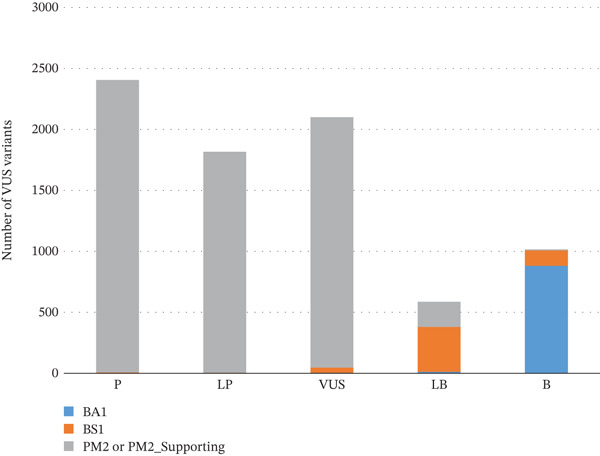
Number of VUS variants incorporating BA1/BS1/PM2.

Variants classified as VUS almost never included BA1/BS1 evidence codes (Figure 3), confirming that high allele frequency is a strong indicator of benignity, while VUS variants lack such definitive population‐based evidence.

### 4.2. Concordance Between Allele Frequency Variant Classification

There was a strong concordance between the application of allele frequency criteria and final variant classification (Table 3). Variants interpreted with BA1 or BS1 criteria were predominantly categorized as B or LB: 99% of BA1‐containing variants and 90.6% of BS1‐containing variants fell into these categories (Table 3). Three variants with BA1 were classified as P, underscoring that the Hearing Loss VCEP is explicitly identified as an exception.

**Table 3:** Consistency between BA1/BS1/PM2 criteria and final variant classification.

Criteria applied	Total number of variants	Proportion of benign (B)	Proportion of likely benign (LB)	Proportion of variant of VUS	Proportion of likely pathogenic (LP)	Proportion of pathogenic (P)
BA1	900	882 (98%)	11 (1.2%)	4 (0.4%)	0	3 (0.3%)
BS1	545	125 (23%)	369 (67.7%)	43 (7.9%)	4 (0.7%)	4 (0.7%)
PM2	6479	9 (0.1%)	207 (3.2%)	2051 (31.7%)	1813 (28%)	2398 (37%)
No BA1/BS1/PM2	1517	34 (2.2%)	373 (24.6%)	747 (49.2%)	131 (8.6%)	232 (15.3%)

Conversely, variants incorporating PM2 criteria were most often classified as P (37%) or LP (30%). This aligns with the role of PM2 as supporting evidence for pathogenicity, as rare variants are more likely to be disease‐causing in the context of other clinical or functional data [1, 20].

## 5. VCEP‐Specific Challenges and Adaptive Strategies

Each VCEP addressed unique clinical and genetic challenges by tailoring threshold parameters and criteria application, as summarized below:

1. Recessive inheritance disorders: VCEPs for recessive disorders (e.g., VLCAD deficiency and GAMT deficiency) allowed for homozygous variants in population databases, as homozygosity may be associated with delayed or mild phenotypes. For example, ACADVL VCEP used a penetrance of 0.75 to account for adult‐onset VLCAD deficiency, while CCDS VCEP assumed 100% penetrance for biallelic variants in GAMT/GATM, reflecting their role as the sole cause of these disorders [25].
2. Rare disorders with unknown prevalence: The BM VCEP faced uncertainty in OCMMPG prevalence (exact frequency unknown) and thus used focal cortical dysplasia (FCD)—the most prevalent linked malformation—as a surrogate. Additionally, PM2 was adjusted to tolerate ≤ 1 gnomAD occurrence to address sequencing errors and the oncogenic nature of AKT3/MTOR/PIK3CA/PIK3R2 (variants enriched in tumor datasets) [6].
3. Oncogenic gene‐related VCEPs (CDH1 and DICER1): These VCEPs implemented strict subpopulation allele count requirements (≥ 5 alleles for BA1/BS1) to avoid false B classifications due to clonal hematopoiesis or cancer dataset contamination. CDH1 VCEP also excluded homozygous variants from PM2 eligibility, as homozygosity for P CDH1 variants is extremely rare in healthy populations [8].
4. Disorders with high genetic heterogeneity (RASopathy and FH): The RASopathy VCEP integrated gene‐specific contributions (e.g., PTPN11 accounts for 50% of Noonan syndrome cases) to refine BS1 thresholds [17], while FH VCEP distinguished between filtering allele frequency (FAF) and minor allele frequency (MAF) for BA1/BS1 and PM2, respectively, to account for variant detection biases in exome versus genome data [10].
5. Disorders with incomplete penetrance (MHS and PTEN): MHS VCEP used a conservative penetrance estimate (0.01) due to the lack of precise penetrance data for MH susceptibility [13], ensuring BA1 thresholds were high enough to avoid misclassifying P variants as B. PTEN VCEP lowered the ACMG/AMP′s original BA1 threshold from 5% to 1% to accommodate variable phenotypic expressivity (e.g., mild PHTS traits often go undiagnosed) [16].
6. X‐linked disorders (Rett/Angelman‐like and CCDS SLC6A8): VCEPs for X‐linked disorders (e.g., SLC6A8‐related CCDS and MECP2‐related Rett syndrome) adjusted thresholds to account for sex‐specific penetrance. For example, CCDS VCEP set SLC6A8 penetrance at 0.5 to reflect asymptomatic female carriers, while Rett/Angelman‐like VCEP noted fewer SLC6A8 carrier females in gnomAD due to potential cognitive/behavioral phenotypes that may exclude them from control cohorts.

## 6. Impact of BA1/BS1/PM2 on Variant Classification (Point‐Based System)

To quantify the specific impact of BA1/BS1/PM2 on variant classification—an aspect not addressed by the categorical ACMG/AMP framework—we applied a Bayesian point–based system developed by Tavtigian et al. (currently refined by the ClinGen SVI Working Group). This system assigns numerical weights to evidence: +1 to +8 for P evidence and −1 to −8 for B evidence, with total scores mapped to final classifications (B, LB, VUS, LP, and P) via predefined cutoffs.

### 6.1. Key Findings From Point‐Based Reclassification

Of 6606 variants with allele frequency data, 299 (4.5%) were reclassified compared to their original ACMG/AMP categorical classification (Table 4).

**Table 4:** Variant reclassification based on Bayesian point–based system.

Original classification	Number of variants with allele frequency	Reclassification direction (to)	Reclassification proportion	Main driving factors
Pathogenic (P)	1773	VUS: 43; LP: 79	2.4% (43/1773); 4.5% (79/1773)	Stable PM2 scores and sufficient other pathogenic evidence
Likely pathogenic (LP)	1463	VUS: 28; P: 24	1.9% (28/1463); 1.6% (24/1463)	Some variants had scores below the LP cutoff; a few were upgraded to P due to the superposition of other evidence
Variant of uncertain significance (VUS)	1685	LP: 5; LB: 61; B: 2	0.3% (5/1685); 3.6% (61/1685); 0.12% (2/1685)	After including PM2, some variants were close to the LP cutoff; or after including BA1/BS1, some were close to the LB cutoff
Likely benign (LB)	635	VUS: 20; B: 22	3.1% (20/635); 3.5% (22/635)	Some variants had scores above the LB cutoff; a few were upgraded to B due to the superposition of other benign evidence
Benign (B)	1050	LB: 15	1.4% (15/1050)	Stable BA1/BS1 scores and sufficient other benign evidence

PM2/PM2_Supporting was critical for P classification: 4211 out of 4585 (91.8%) P/LP variants relied on PM2 for their final classification (Table 3). When PM2 points were removed, 747 out of 1813 (41%) LP variants were downgraded to VUS, highlighting PM2′s role as a “threshold” criterion for distinguishing LP from VUS.

BA1/BS1 had a larger and consistent impact on B classification: 302 out of 380 (79.5%) LB variants were reclassified as VUS when BA1/BS1 points were excluded (Figure 1), confirming that high allele frequency strengthens B evidence [20].

The point‐based system revealed borderline variants that the categorical ACMG/AMP framework failed to distinguish. For example, 198 variants classified as VUS via ACMG/AMP scored just below the LP cutoff when PM2 was included, indicating their classification was highly dependent on allele frequency evidence.

### 6.2. Cross‐VCEP Consistency

Reclassified variants were distributed across all VCEPs (Table 4), with no single panel accounting for more than 5% of reclassifications. This indicates that most VCEPs place comparable emphasis on allele frequency criteria, despite variations in threshold parameters. The consistency in impact across disease groups suggests that BA1/BS1/PM2 serve as universal, cross‐disorder tools for variant interpretation, with their influence modulated by disorder‐specific parameters (e.g., prevalence and penetrance).

## 7. Summary of Threshold‐Setting Approaches

VCEPs employed three primary strategies to establish BA1/BS1/PM2 thresholds, reflecting the balance between generalizability and disorder specificity:

1. Conservative parameter selection: Most VCEPs used conservative estimates for prevalence (e.g., ACADVL′s 1/30,000 for VLCADD) and penetrance (e.g., MHS′s 0.01) to avoid false classification of P variants as B.
2. Data‐driven adjustments: Thresholds were refined using variant‐specific data (e.g., ACADVL′s 0.2 allelic contribution based on c.848T>C frequency) or population database validation (e.g., CDH1′s PM2 threshold validated with rare LoF variants in gnomAD).
3. Framework adaptation: VCEPs modified the original ACMG/AMP criteria to address unique challenges (e.g., BM VCEP′s PM2 adjustment for cancer datasets and PTEN VCEP′s BA1 threshold reduction).

These strategies collectively ensure that allele frequency thresholds are both evidence‐based and clinically relevant while acknowledging the inherent variability in genetic disorders′ epidemiology and molecular mechanisms.

## 8. Discussion

Our analysis of 39 VCEPs encompassing a broad spectrum of Mendelian disorders—ranging from hereditary cancers (BRCA2 and TP53) and neurodevelopmental conditions (SCN2A and RPGR) to metabolic diseases (GCK and ACADVL) and rare myopathies (ACTA1 and SGCB)—unveils a critical paradox: While gene/disease‐specific adaptation of ACMG/AMP guidelines has enhanced variant interpretation precision, inconsistent parameterization, validation methodologies, and allele frequency data utilization undermine cross‐panel reproducibility. This synthesis highlights the urgent need for targeted consensus frameworks to reconcile specificity with standardization, ultimately strengthening the clinical utility of variant classification across diverse disease entities.

### 8.1. Parameter Specification in Whiffin–Ware Calculator: Documented Evidence Versus Extrapolation

Across the 39 VCEPs, parameter specification for the Whiffin–Ware calculator fell into two distinct categories, reflecting disease‐specific data availability. A majority of 25 VCEPs (62.5%) utilized fully documented parameters, anchored in robust population cohorts, clinical registries, or functional assay data [18]. For example, the ABCA4 VCEP (autosomal recessive retinopathy) integrated a validated prevalence of 1 in 7500 and tissue‐specific penetrance data from retinal organoid models, while the GCK VCEP (monogenic diabetes) calibrated inputs using glucose tolerance test outcomes and variant‐specific functional impact scores. The MYOC VCEP (primary open‐angle glaucoma) further refined parameters with population‐based penetrance estimates (56% for p.Gln368Ter) and longitudinal disease progression data, ensuring calculator outputs aligned with real‐world clinical observations. Similarly, the BRCA2 VCEP leveraged large‐scale cancer registry data to define allelic contribution thresholds for P missense variants in the RING domain.

In contrast, 15 VCEPs (37.5%) resorted to extrapolation due to limited evidence, employing surrogate metrics or conservative assumptions to address data gaps. The SLC6A8 VCEP (X‐linked creatine transporter deficiency) used plasma creatine/creatinine ratios as a proxy for disease prevalence, while the RYR1 VCEP (congenital myopathy) adopted a universal penetrance estimate of 0.5 for autosomal recessive variants. The MSH2 VCEP (Lynch syndrome) extrapolated allelic contribution from tumor microsatellite instability data, and the ENG VCEP (hereditary hemorrhagic telangiectasia) relied on cysteine residue conservation to infer functional impact for splice site variants. Novel examples from expanded VCEPs include the AIPL1 VCEP (Leber congenital amaurosis), which used photoreceptor cell–type expression data to extrapolate allelic contribution, and the SERPINC1 VCEP (antithrombin deficiency), which employed plasma antithrombin activity levels as a surrogate for penetrance. This distribution underscores a core challenge: While most VCEPs can leverage well‐characterized parameters, nearly two‐fifths face unavoidable subjectivity—emphasizing the need for ClinGen to develop standardized extrapolation frameworks, particularly for ultrarare diseases with limited epidemiological data.

### 8.2. Heterogeneity in Threshold Setting: Manifestations and Underlying Drivers

#### 8.2.1. Core Patterns of Threshold Variability

Thresholds for BA1, BS1, and PM2 span multiple orders of magnitude across VCEPs, with variability systematically linked to disease biology and inheritance patterns.

For BA1, the original ACMG/AMP guideline′s 5% (0.05) AF threshold is rarely applied directly. Instead, VCEPs tailor thresholds to disease prevalence and penetrance: The MYOC VCEP (autosomal dominant open‐angle glaucoma) sets BA1 at 1% (0.01) to account for late onset and incomplete penetrance, while the CDH1 VCEP (hereditary diffuse gastric cancer) adopts a stricter 0.2% (0.002) due to the disease′s rarity and high penetrance. X‐linked disorders further refine BA1 by integrating hemizygote counts: The SLC6A8 VCEP (creatine transporter deficiency) defines BA1 as AF > 0.2*%* (0.002) or ≥ 10 hemizygotes in gnomAD, recognizing the unique dosage dynamics of X‐linked inheritance [18].

BS1 thresholds similarly reflect disease‐specific AF expectations. Autosomal recessive disorders, requiring biallelic P variants, tolerate higher BS1 thresholds: The Hearing Loss VCEP sets BS1 at 0.3% (0.003) for recessive forms, while dominant disorders like FBN1‐related Marfan syndrome use a stricter 0.005% (0.00005) to avoid misclassifying rare P variants. Cancer predisposition genes (e.g., BRCA2) further tighten BS1 to 0.01% (0.0001) by leveraging disease‐excluded databases (gnomAD noncancer subsets) to minimize confounding from undiagnosed cases [11, 23].

PM2 thresholds emphasize rarity, with recessive diseases requiring extremely low AF to account for carrier frequencies: The ACTA1 VCEP (autosomal recessive *α*‐actinopathy) sets PM2 at ≤ 0.00005 (0.005%), while the RYR1 VCEP (autosomal recessive myopathy) uses an even stricter ≤ 0.00000697 (0.000697%). Dominant diseases (e.g., SCN2A‐related neurodevelopmental disorder) adopt moderately higher PM2 thresholds (≤ 0.0001, 0.01%), balancing rarity with the higher impact of single‐allele pathogenicity. X‐linked and cancer‐related VCEPs add layers of refinement: The F9 VCEP (Hemophilia B, X‐linked) requires PM2 variants to be absent in males in gnomAD, while BRCA2 excludes insertion/deletion variants from PM2 due to poor AF calling in population databases [15, 18].

#### 8.2.2. Key Drivers of Threshold Heterogeneity

Inheritance pattern and disease prevalence: Autosomal recessive diseases demand higher PM2 thresholds (to account for carrier frequencies) and BS1 thresholds (to tolerate B carrier variants), while dominant diseases prioritize stricter BS1 to limit false B calls. Rare diseases (e.g., CDH1‐related gastric cancer, prevalence ~1/1,000,000) use tighter BA1/BS1 thresholds than more common conditions (e.g., MYOC‐related glaucoma, prevalence ~1/2000).

Population database selection: Only 17.5% of VCEPs (e.g., BRCA2 and PMS2) use disease‐excluded databases (gnomAD noncancer, disease‐specific cohorts), while 82.5% rely on general population databases (full gnomAD). This choice directly impacts thresholds: BRCA2′s BS1 threshold of 0.01% (0.0001) is calibrated to gnomAD′s noncancer subset, reducing bias from undiagnosed cancer cases that would inflate AF in general databases.

Penetrance and phenotypic heterogeneity: Diseases with incomplete penetrance (e.g., MYOC glaucoma) or variable expressivity (e.g., SCN2A neurodevelopmental disorder) adopt more lenient BA1/BS1 thresholds to avoid misclassifying variants present in asymptomatic carriers. Conversely, fully penetrant diseases (e.g., CDH1 gastric cancer) use stricter thresholds to prioritize sensitivity for P variants.

Variant type and technical considerations: Some VCEPs exclude specific variant types from PM2 (e.g., BRCA2 indels and MSH2 initiation codon variants) due to poor AF calling or unique functional constraints, further narrowing the scope of AF‐based criteria [1, 21, 23].

### 8.3. Clinical Challenges Posed by Threshold Heterogeneity

#### 8.3.1. Compromised Reproducibility and Interlaboratory Consistency

Inconsistent thresholds lead to divergent classification of identical variants across VCEPs. For example, a variant with an AF of 0.05% (0.0005) would meet BS1 (B supporting) in the CDH1 VCEP (BS1 threshold 0.1%) but not in the SLC6A8 VCEP (BS1 threshold 0.02%), creating ambiguity for variants in genes linked to multiple diseases or when data is shared across clinical laboratories. This inconsistency is particularly problematic for multigene panel testing, where variants may be evaluated against VCEP‐specific thresholds without explicit transparency to clinicians.

#### 8.3.2. Ambiguity in Threshold Validation and Extrapolation

Nearly 37.5% of VCEPs extrapolate thresholds using surrogate data due to limited disease‐specific AF resources. The SLC6A8 VCEP uses plasma creatine/creatinine ratio as a proxy for disease prevalence to calibrate BS1, while the RYR1 VCEP relies on general penetrance estimates for recessive disease. Additionally, 12.5% of VCEPs do not specify formal validation methods, relying solely on computational calculator outputs (e.g., Whiffen–Ware) without empirical validation against clinical cohorts. This lack of rigorous validation increases the risk of thresholds misaligning with real‐world variant pathogenicity.

#### 8.3.3. Underutilization of Disease‐Excluded Databases and Quality Controls

Most VCEPs (82.5%) rely on general population databases, which are contaminated by undiagnosed late‐onset diseases (e.g., FBN1‐related thoracic aortic disease) or subclinical carriers. This contamination can inflate AF for rare P variants, leading to false BS1/BA1 calls. Furthermore, only a subset of VCEPs (e.g., BRCA2 and CDH1) incorporates quality control filters (e.g., minimum read depth ≥ 20 and exclusion of bottlenecked populations) when setting thresholds, increasing vulnerability to artifacts in population AF data.

### 8.4. Toward Balanced Standardization: Principles and Practical Recommendations

The goal of standardization is not to enforce a one‐size‐fits‐all threshold but to establish transparent, evidence‐based frameworks for calibrating AF‐driven criteria—preserving gene/disease specificity while ensuring reproducibility.

#### 8.4.1. Guiding Principles for Threshold Calibration

Disease biology‐centric calibration: Thresholds should be anchored in disease prevalence, inheritance pattern, and penetrance. For autosomal recessive diseases, PM2 thresholds should account for carrier frequency, while dominant diseases should link BS1 to the maximum credible AF for P variants (derived from epidemiological data) [4, 12].

Database‐specific thresholds: VCEPs should explicitly specify the population database (general vs. disease‐excluded) and version used for threshold calibration. Disease‐excluded databases should be prioritized for late‐onset or cancer predisposition genes to reduce confounding, with thresholds adjusted to account for database‐specific sample sizes and demographics [11, 23].

Validation and transparency: Thresholds must be validated against clinical cohorts (e.g., case–control AF comparisons) or functional data. VCEPs should document validation methods (e.g., Whiffen–Ware calculator parameters and empirical AF distributions in cases) and make threshold rationales publicly accessible to facilitate cross‐VCEP comparison [8].

#### 8.4.2. Practical Recommendations for Standardization

Establish reference threshold frameworks by disease class: Develop class‐specific default thresholds (e.g., autosomal dominant vs. recessive and cancer vs. pediatric) to reduce variability while allowing gene‐specific adjustments [4, 11].

For example

Autosomal dominant diseases: BA1 ≥ 1*%*, BS1 ≥ 0.1*%*, and PM2 ≤ 0.0001 (general database)

Autosomal recessive diseases: BA1 ≥ 0.5*%*, BS1 ≥ 0.3*%*, and PM2 ≤ 0.00005 (general database)

Cancer predisposition genes: BA1 ≥ 0.1*%*, BS1 ≥ 0.01*%*, and PM2 ≤ 0.00001 (disease‐excluded database)

Incorporate dosage and inheritance‐specific adjustments: For X‐linked genes, thresholds should integrate hemizygote counts (e.g., BS1 ≥ 5 hemizygotes) alongside AF. For mitochondrial genes, account for heteroplasmy levels (e.g., PM2 < 0.00002 AF for homoplasmic variants).

Mandate quality control for AF data: Require VCEPs to specify minimum read depth (≥ 25), sample size (≥ 2000 alleles), and exclusion of bottlenecked populations (e.g., Finnish and Ashkenazi Jewish) when setting thresholds, reducing artifacts from poor‐quality AF data.

Develop a centralized threshold repository: Curate VCEP‐specific thresholds for BA1/BS1/PM2 in a searchable database, linked to disease epidemiology, database source, and validation methods. This resource would enable clinical laboratories to align with VCEP standards and facilitate cross‐gene comparisons.

## 9. Supplemented for BA1 Criterion Clarification

In variant interpretation, the application of evidence criteria strictly follows standardized guidelines to ensure the consistency and reliability of classification results. Among these criteria, BA1 is universally recognized as a standalone criterion for B classification. Specifically, once a variant meets the BA1 criterion (e.g., allele frequency significantly exceeding the disease prevalence, supporting its B nature), it should be directly classified as B, and thus should not be categorized as a VUS, LP, or P by definition. This rule is widely adopted in most variant interpretation practices and VCEP annotations, which is also consistent with our observation that VUS rarely include variants meeting BA1.

Notably, an exception was observed in the annotation results of the ACADVL VCEP, Mitochondrial Disease Nuclear and Mitochondrial VCEP (four variants annotated with the BA1 criterion were ultimately classified as VUS), and Hearing Loss VCEP (three variants annotated with the BA1 criterion were ultimately classified as P). This finding deviates from the general application rule of BA1 but is strictly based on the official annotation records of the ACADVL VCEP, Mitochondrial Disease Nuclear and Mitochondrial VCEP, and Hearing Loss VCEP. We speculate that this specific annotation practice may be related to the unique characteristics of those diseases (e.g., rare subtypes with unclear prevalence, potential phenotypic heterogeneity, or special genetic backgrounds) or the VCEPs′ additional consideration of other unreported evidence during the curation process. Although the detailed rationale for this annotation has not been explicitly stated in the VCEPs′ official documentation, we have retained the original classification results in this study to ensure the authenticity and traceability of the data, with corresponding explanatory notes added in Table 3. Hearing loss exhibits marked allelic heterogeneity and phenotypic heterogeneity—key genetic features that explain the BA1‐P discrepancy. For the same gene, different variants may exert a broad spectrum of effects (from B to P) due to variation in functional impact, genetic background, or environmental modifiers. These characteristics mean that a variant′s high AF (meeting BA1) does not inherently rule out pathogenicity, especially for autosomal recessive hearing loss—where carriers (heterozygotes) with high AF are common in the general population, but homozygotes or compound heterozygotes still develop severe disease.

This exceptional case highlights the need for caution when applying general interpretation guidelines to specific disease‐related VCEP annotations. For researchers engaged in variant curation of ACADVL, Mitochondrial Disease, Hearing Loss, or similar rare diseases, it is necessary to fully understand the specific criteria and annotation logic of the corresponding VCEP to avoid misinterpretation of results. In future studies, we recommend further communication with the ACADVL VCEP, Mitochondrial Disease, and Hearing Loss VCEPs to clarify the underlying reasons for this special annotation practice, which will help optimize the consistency of variant interpretation criteria across different diseases and improve the accuracy of clinical genetic diagnosis.

BA1, BS1, and PM2 are indispensable for translating population AF data into clinically actionable variant classifications, but their heterogeneity across VCEPs highlights the tension between gene/disease specificity and standardization. By anchoring thresholds in disease biology, prioritizing validated databases, and establishing transparent class‐specific frameworks, we can reduce unnecessary variability while preserving the flexibility to adapt to unique gene/disease characteristics. Such balanced standardization will enhance the reproducibility of variant interpretation, improve clinical confidence in genetic testing results, and ultimately advance the utility of genomic medicine for patient care [5, 24].

## Author Contributions

Jieping Song and Xiaoyan Wang were instrumental in devising and planning the study. Xiaoyan Wang, along with Tongmei Zhang, was responsible for acquiring and categorizing the data. The analytical work was conducted by Xiaoyan Wang and Yayun Qin. The first draft of the manuscript was authored by Xiaoyan Wang, Jieping Song, Yongyi Zou, Haiyan Luo, Haibo Li, and Tongmei Zhang. Subsequent revisions and finalization of the manuscript were a collaborative effort among all listed authors.

## Funding

No funding was received for this manuscript.

## Ethics Statement

The individual supplying the data verifies that they possess all required permissions to disseminate the information to ClinGen and consents to its utilization in accordance with the present description.

## Conflicts of Interest

The authors declare no conflicts of interest.

## Data Availability

The data sets that were either created or examined for this research can be accessed through the ClinGen Variant Curation Interface, which is located at https://clinicalgenome.org/tools/educational-resources/variant-interpretation-topics/variant-curation-interface/.

## References

[1] Richards S. , Aziz N. , Bale S. , Bick D. , Das S. , Gastier-Foster J. , Grody W. W. , Hegde M. , Lyon E. , Spector E. , and Voelkerding K. , Standards and Guidelines for the Interpretation of Sequence Variants: A Joint Consensus Recommendation of the American College of Medical Genetics and Genomics and the Association for Molecular Pathology, Genetics in Medicine. (2015) 17, no. 5, 405–423, 10.1038/gim.2015.30, 2-s2.0-84928209346. 25741868 PMC4544753

[2] Brnich S. E. , Rivera-Mu Oz E. A. , and Berg J. S. , Quantifying the Potential of Functional Evidence to Reclassify Variants of Uncertain Significance in the Categorical and Bayesian Interpretation Frameworks, Human Mutation. (2018) 39, no. 11, 1531–1541, 10.1002/humu.23609, 2-s2.0-85052914746. 30095857 PMC6548460

[3] Kanavy D. M. , McNulty S. M. , Jairath M. K. , Brnich S. E. , Bizon C. , Powell B. C. , and Berg J. S. , Comparative Analysis of Functional Assay Evidence Use by ClinGen Variant Curation Expert Panels, Genome Medicine. (2019) 11, no. 1, 10.1186/s13073-019-0683-1. PMC6884856 31783775

[4] Rivera‐Muñoz E. A. , Milko L. V. , Harrison S. M. , Azzariti D. R. , Kurtz C. L. , Lee K. , Mester J. L. , Weaver M. A. , Currey E. , Craigen W. , and Eng C. , ClinGen Variant Curation Expert Panel Experiences and Standardized Processes for Disease and Gene-Level Specification of the ACMG/AMP Guidelines for Sequence Variant Interpretation, Human Mutation. (2018) 39, no. 11, 1614–1622, 10.1002/humu.23645, 2-s2.0-85054701150. 30311389 PMC6225902

[5] Flowers M. , Dickson A. , Miller M. J. , Spector E. , Enns G. M. , and Baudet H. , Specifications of the ACMG/AMP Guidelines for ACADVL Variant Interpretation, Molecular Genetics and Metabolism. (2023) 140, no. 3, 107668, 10.1016/j.ymgme.2023.107668. 37549443 PMC10811274

[6] Lai A. , Soucy A. , El Achkar C. M. , Barkovich A. J. , Cao Y. , and DiStefano M. , The ClinGen Brain Malformation Variant Curation Expert Panel: Rules for Somatic Variants in AKT3, MTOR, PIK3CA, and PIK3R2, Genetics in Medicine. (2022) 24, no. 11, 2240–2248, 10.1016/j.gim.2022.07.020. 35997716 PMC9883838

[7] Kelly M. A. , Caleshu C. , Morales A. , Buchan J. , Wolf Z. , and Harrison S. M. , Adaptation and Validation of the ACMG/AMP Variant Classification Framework for MYH7-Associated Inherited Cardiomyopathies: Recommendations by ClinGen′s Inherited Cardiomyopathy Expert Panel, Genetics in Medicine. (2018) 20, no. 3, 351–359, 10.1038/gim.2017.218, 2-s2.0-85044572143. 29300372 PMC5876064

[8] Lee K. , Krempely K. , Roberts M. E. , Anderson M. J. , Carneiro F. , and Chao E. , Specifications of the ACMG/AMP Variant Curation Guidelines for the Analysis of Germline CDH1 Sequence Variants, Human Mutation. (2018) 39, no. 11, 1553–1568, 10.1002/humu.23650, 2-s2.0-85054665075. 30311375 PMC6188664

[9] Hatton J. N. , Frone M. N. , Cox H. C. , Crowley S. B. , Hiraki S. , and Yokoyama N. N. , Specifications of the ACMG/AMP Variant Classification Guidelines for Germline DICER1 Variant Curation, Human Mutation. (2023) 2023, 9537832, 10.1155/2023/9537832. 38084291 PMC10713350

[10] Chora J. R. , Iacocca M. A. , Tichy L. , Wand H. , Kurtz C. L. , and Zimmermann H. , The Clinical Genome Resource (ClinGen) Familial Hypercholesterolemia Variant Curation Expert Panel Consensus Guidelines for LDLR Variant Classification, Genetics in Medicine. (2022) 24, no. 2, 293–306, 10.1016/j.gim.2021.09.012. 34906454 PMC12558601

[11] Spier I. , Yin X. , Richardson M. , Pineda M. , Laner A. , and Ritter D. , Gene-Specific ACMG/AMP Classification Criteria for Germline APC Variants: Recommendations From the ClinGen InSiGHT Hereditary Colorectal Cancer/Polyposis Variant Curation Expert Panel, Genetics in Medicine. (2024) 26, no. 2, 100992, 10.1016/j.gim.2023.100992. 37800450 PMC10922469

[12] Goldstein J. L. , McGlaughon J. , Kanavy D. , Goomber S. , Pan Y. , and Deml B. , Variant Classification for Pompe Disease; ACMG/AMP Specifications From the ClinGen Lysosomal Diseases Variant Curation Expert Panel, Molecular Genetics and Metabolism. (2023) 140, no. 1-2, 107715, 10.1016/j.ymgme.2023.107715. 37907381 PMC10872922

[13] Johnston J. J. , Dirksen R. T. , Girard T. , Gonsalves S. G. , Hopkins P. M. , and Riazi S. , Variant Curation Expert Panel Recommendations for RYR1 Pathogenicity Classifications in Malignant Hyperthermia Susceptibility, Genetics in Medicine. (2021) 23, no. 7, 1288–1295, 10.1038/s41436-021-01125-w. 33767344 PMC8263483

[14] Zastrow D. B. , Baudet H. , Shen W. , Thomas A. , Si Y. , and Weaver M. A. , Unique Aspects of Sequence Variant Interpretation for Inborn Errors of Metabolism (IEM): The ClinGen IEM Working Group and the Phenylalanine Hydroxylase Gene, Human Mutation. (2018) 39, no. 11, 1569–1580, 10.1002/humu.23649, 2-s2.0-85054685693. 30311390 PMC6556116

[15] Ross J. E. , Zhang B. M. , Lee K. , Mohan S. , Branchford B. R. , Bray P. , Dugan S. N. , Freson K. , Heller P. G. , Kahr W. H. , and Lambert M. P. , Specifications of the Variant Curation Guidelines for ITGA2B/ITGB3 ClinGen Platelet Disorder Variant Curation Panel, Blood Advances. (2021) 5, no. 2, 414–431, 10.1182/bloodadvances.2020003712. 33496739 PMC7839359

[16] Mester J. L. , Ghosh R. , Pesaran T. , Huether R. , Karam R. , Hruska K. S. , Costa H. A. , Lachlan K. , Ngeow J. , Barnholtz-Sloan J. , and Sesock K. , Gene-Specific Criteria for PTEN Variant Curation: Recommendations From the ClinGen PTEN Expert Panel, Human Mutation. (2018) 39, no. 11, 1581–1592, 10.1002/humu.23636, 2-s2.0-85054693817. 30311380 PMC6329583

[17] Gelb B. D. , Cavé H. , Dillon M. W. , Gripp K. W. , Lee J. A. , Mason-Suares H. , Rauen K. A. , Williams B. , Zenker M. , and Vincent L. M. , ClinGen′s RASopathy Expert Panel Consensus Methods for Variant Interpretation, Genetics in Medicine. (2018) 20, no. 11, 1334–1345, 10.1038/gim.2018.3, 2-s2.0-85054690075. 29493581 PMC6119537

[18] McKnight D. , Bean L. , Karbassi I. , Beattie K. , Bienvenu T. , Bonin H. , Fang P. , Chrisodoulou J. , Friez M. , Helgeson M. , and Krishnaraj R. , Recommendations by the ClinGen Rett/Angelman-Like Expert Panel for Gene-Specific Variant Interpretation Methods, Human Mutation. (2022) 43, no. 8, 1097–1113, 10.1002/humu.24302. 34837432 PMC9135956

[19] Whiffin N. , Minikel E. , Walsh R. , O’Donnell-Luria A. H. , Karczewski K. , Ing A. Y. , Barton P. J. , Funke B. , Cook S. A. , MacArthur D. , and Ware J. S. , Using High-Resolution Variant Frequencies to Empower Clinical Genome Interpretation, Genetics in Medicine. (2017) 19, no. 10, 1151–1158, 10.1038/gim.2017.26, 2-s2.0-85028908595. 28518168 PMC5563454

[20] Tavtigian S. V. , Greenblatt M. S. , Harrison S. M. , Nussbaum R. L. , Prabhu S. A. , Boucher K. M. , and Biesecker L. G. , Modeling the ACMG/AMP Variant Classification Guidelines as a Bayesian Classification framework, Genetics in Medicine. (2018) 20, no. 9, 1054–1060, 10.1038/gim.2017.210, 2-s2.0-85049273706. 29300386 PMC6336098

[21] Ibarra Moreno C. A. , Hu S. , Kraeva N. , Schuster F. , Johannsen S. , Rueffert H. , Klingler W. , Heytens L. , and Riazi S. , An Assessment of Penetrance and Clinical Expression of Malignant Hyperthermia in Individuals Carrying Diagnostic Ryanodine Receptor 1 Gene Mutations, Anesthesiology. (2019) 131, no. 5, 983–991, 10.1097/ALN.0000000000002813, 2-s2.0-85073305963, 31206373. 31206373 PMC9912949

[22] Reuser A. J. J. , van der Ploeg A. T. , Chien Y. , Llerena J. J. , Abbott M. , Clemens P. R. , Kimonis V. E. , Leslie N. , Maruti S. S. , Sanson B. J. , Araujo R. , Periquet M. , Toscano A. , Kishnani P. S. , and Pompe Registry Sit , GAA Variants and Phenotypes Among 1,079 Patients With Pompe Disease: Data From the Pompe Registry, Human Mutation. (2019) 40, no. 11, 2146–2164, 10.1002/humu.23878, 2-s2.0-85070684264, 31342611. 31342611 PMC6852536

[23] Lek M. , Karczewski K. J. , Minikel E. V. , Samocha K. E. , Banks E. , Fennell T. , O’Donnell-Luria A. H. , Ware J. S. , Hill A. J. , Cummings B. B. , and Tukiainen T. , Analysis of Protein-Coding Genetic Variation in 60,706 Humans, Nature. (2016) 536, no. 7616, 285–291, 10.1038/nature19057, 2-s2.0-84982253941, 27535533. 27535533 PMC5018207

[24] Ghosh R. , Harrison S. M. , Rehm H. L. , Plon S. E. , Biesecker L. G. , and ClinGen S. V. I. W. , Updated Recommendation for the Benign Stand-Alone ACMG/AMP Criterion, Human Mutation. (2018) 39, no. 11, 1525–1530, 10.1002/humu.23642, 2-s2.0-85054606074, 30311383. 30311383 PMC6188666

[25] Salomons G. S. , van Dooren S. J. , Verhoeven N. M. , Cecil K. M. , Ball W. S. , Degrauw T. J. , and Jakobs C. , X-Linked Creatine-Transporter Gene (SLC6A8) Defect: A New Creatine-Deficiency Syndrome, American Journal of Human Genetics. (2001) 68, no. 6, 1497–1500, 10.1086/320595, 2-s2.0-0034987448, 11326334. 11326334 PMC1226136

